# Interventions to reduce inequalities for pregnant women living with disadvantage in high-income countries: an umbrella review protocol

**DOI:** 10.1186/s13643-024-02556-7

**Published:** 2024-05-23

**Authors:** N. Vousden, D. Geddes-Barton, N. Roberts, M. Knight

**Affiliations:** 1 Department of Population Health, National Perinatal Epidemiology Unit, University of Oxford, Old Road Campus, NuffieldHeadington, Oxford OX3 7LF UK; 2 Nuffield Department of Population Health, Nuffield Department of Primary Care Health Sciences and Department of Oncology, Bodleian Health Care Libraries, Oxford, UK

**Keywords:** Pregnancy, Inequalities, Disadvantage, Deprivation, Social risk, Umbrella review, Effectiveness

## Abstract

**Background:**

Women who live with disadvantages such as socioeconomic deprivation, substance misuse, poor mental health, or domestic abuse face inequalities in health before, during, and after pregnancy and for their infants through to childhood. Women do not experience these factors alone; they accumulate and interact. Therefore, there is a need for an overview of interventions that work across health and social care and target women at risk of inequalities in maternal or child health.

**Methods:**

Systematic review methodology will be used to identify systematic reviews from high-income countries that describe interventions aiming to reduce inequalities for women who experience social disadvantage during pregnancy. We will describe the range of interventions and their effectiveness in reducing inequalities in maternal or child health. Any individual, hospital, or community-level activity specific to women during the pre-conception, antenatal, or postpartum period up to 1 year after birth will be included, regardless of the setting in which they are delivered. We will search eight electronic databases with the pre-determined search strategy and supplement them with extensive grey literature searches. We will present a narrative synthesis, taking into account the quality assessment and coverage of included studies.

**Discussion:**

Inequalities in maternal and child health are a key priority area for national policymakers. Understanding the range and effectiveness of interventions across the perinatal period will inform policy and practice. Identifying gaps in the evidence will inform future research.

**Systematic review registration:**

PROSPERO CRD42023455502.

**Supplementary Information:**

The online version contains supplementary material available at 10.1186/s13643-024-02556-7.

## Background

Women who live in the most deprived areas of England are more than twice as likely to die in pregnancy [1] or experience poor outcomes such as stillbirth [2], preterm birth, and fetal growth restriction [3] compared to women in the most affluent areas. This evidence is based on neighborhood deprivation measures therefore inadequately describes the extent of health inequity experienced by individuals who live at the extreme margins of social disadvantage or have multiple intersecting risk factors. For example, low socioeconomic status is often associated with increased social risks such as housing instability and poor mental health. Confidential inquiries into the care of women who die during pregnancy and the postnatal period in the United Kingdom have identified that 12% experienced severe and multiple disadvantages [1], most commonly mental health diagnosis, substance use, and domestic abuse [1]. In addition, the negative health and educational impacts on offspring of growing up in poverty [4] or being exposed to adverse experiences in childhood [5] are widely documented. This highlights the need for interventions to effectively prevent and address multiple complex needs as a whole, rather than for discrete populations with a single risk factor.

Reducing socio-economic inequalities in maternal and perinatal health has been a key priority for the government in the UK [6–8] and internationally [9]. Current National Institute for Health and Care Excellence (NICE) guidance on complex social factors in pregnancy, written in 2010, focuses on four key areas, women with alcohol or drug misuse, recent migrant or asylum seeker status, young mothers, and women experiencing domestic abuse [10]. However, it was written in 2010 and the lack of high-quality evidence to inform personalized care for women with social complexity, meant the guidance heavily relied on expert opinion. Later reviews of this guidance in 2012 and 2018 recommended a wider range of social complexity was covered including mental health and homelessness [11].

There are conflicting opinions on the persisting focus on health systems to mitigate social determinants of health. While structural change, for example, reduction of family poverty through improved welfare systems and employment opportunities is undoubtedly vital [12], pregnancy and the postnatal period have been proposed to be a unique opportunity when nearly all women access health services and potentially can be engaged in health-promoting activities with long-term intergenerational impacts [13]. Existing reviews have explored interventions to reduce inequalities focusing on the model of antenatal care [14, 15] or specific short-term pregnancy outcomes such as preterm birth [16]. But, given that socioeconomic disadvantage is present before pregnancy, and has far-reaching implications for the next generation, there is a need to identify and understand interventions that utilize a cross-disciplinary approach, beyond maternity care [17].

Confidential inquiries into maternal mortality in the UK consistently report the need for improved integration of health and social care services to create seamless pathways [17]. This is echoed in an analysis of the Lancet series on midwifery, which highlighted that future research should prioritize care “tailored to individuals, (which) weighs benefits and harms, is person-centered, (and) works across the whole continuum of care” [7]. Therefore, there is a need for an overview of interventions across health and social care targeting women at risk of inequalities in maternal or child health as a result of social disadvantage before, during, and after pregnancy.

This review is informed by perspectives on social exclusion [18], intersectionality [19], and life-course epidemiology [20] which examine how factors accumulate and intersect over time and affect health. With a recent policy that prioritizes person-centered care [7] and equity of health outcomes [7, 21], we anticipate that the findings will be beneficial to policymakers, service providers, and commissioners.

## Methods

### Registration and protocol adherence

This research question has been developed by a multidisciplinary team of clinicians, researchers, and methodologists. It was designed in accordance with Preferred Reporting Items for Systematic Reviews and Meta-Analysis Protocols (PRISMA) [22] and registered on the International Prospective Register of Systematic Reviews on 22/08/2023 (PROSPERO, no: CRD42023455502). We started developing this protocol in May 2023 and the review is expected to be completed in January 2024. The terms ‘pregnant women’ and ‘mothers’ will be used throughout this paper [23], but the authors recognize not everyone who is pregnant or giving birth will identify as a woman or a mother.

### Objectives

This systematic review aims to synthesize the quantitative literature in order to identify what interventions exist, and how effective they are, at reducing inequalities in maternal and child health for pregnant women living with disadvantages in high-income countries.

### Eligibility Criteria and Population, Intervention, Comparison, and Outcome (PICO) framework

#### Population

There is no clear definition for risks that define disadvantage. Therefore, the eligibility criteria for this study were defined as exposures or factors that are associated with deprivation and with adverse maternal or fetal outcomes (or a strong hypothesis for this, in the absence of data). Women with one or more of the following exposures prior to or during pregnancy: substance or alcohol misuse (excluding tobacco), involvement in the judicial system/prison, victim of modern slavery, homelessness or insecure housing, socioeconomic deprivation (measured for individual), domestic abuse or intimate partner violence, experience of sex work, young mothers (age < 20) [24–26], underserved migrant women, women with a mental health diagnosis [27] and women from minoritized ethnic groups including Gypsy, Roma and Travelling communities [28].

#### Interventions

Any individual, hospital, or community-level activity will be included, regardless of the setting in which they are delivered. Interventions must be specific to women during the pre-conception, antenatal, or postpartum period up to 1 year after birth. For the purposes of this paper, we defined pre-conception as interventions that targeted preparing for a healthy pregnancy. Therefore, interventions in women of reproductive age who experienced disadvantages that were not targeting any part of the pregnancy journey (preparing, pregnancy, or postnatal period) will not be eligible for inclusion. We will also evaluate the suitability of any effective interventions to the UK health and social care setting and perform a sub-analysis if appropriate.

#### Control

Studies with any comparison or control group will be included.

#### Outcome

The main outcomes of interest will be maternal morbidity and mortality, preterm birth, birth weight, and attendance at care, reflecting the existing literature on important inequalities in maternal and child health. However, we will include all outcomes related to inequalities in maternal and child health up to 5 years of age from all retrieved studies. For example, mode of birth, mental well-being as assessed by validated screening scales, breastfeeding initiation and duration, family planning, immunization, and indicators of adverse childhood experience (e.g., emergency hospital attendances).

#### Inclusion and exclusion criteria

This umbrella review will include only systematic reviews and meta-analyses as defined by the Cochrane Collaboration’s Handbook definition of systematic reviews of interventions (i.e., “reviews of clearly formulated questions that use systematic and explicit methods to identify, select and critically appraise relevant research”). Therefore, the reviews must use a comprehensive literature search strategy and a satisfactory technique for assessing the risk of bias in individual studies that were included in the review. This has been chosen as the aim is to assess the effectiveness of interventions, not just identify or map interventions, and therefore assessment of methodological limitations and bias is the key in the interpretation of the evidence. The umbrella review will enable a summation of a broad literature base of disadvantages in pregnancy which would be unfeasible if primary studies were included.

Studies undertaken in high-income countries, as defined by the World Bank GNI (2019), published in any language from 2013 to 2023 will be included. This 10-year period was selected as per the Joanna Briggs Institute guidance as they are considered to represent the contemporaneous evidence base over the previous 30 years [29]. Included studies will either specifically target the defined population or present disaggregated data for a defined subgroup. Abstracts, comments, editorials, letters, or non-systematic reviews will be excluded. Solely qualitative studies will be excluded. We will include systematic reviews of randomized controlled trials and non-randomized studies of health care interventions. This method has been selected as we aspire to identify the range of interventions available across the spectrum of disadvantage, but also be able to report definitive effectiveness data with a feasible number of results.

#### Data collection and appraisal

The search strategy has been developed with assistance from an expert information specialist and adapted for each database (Additional file 1). The following electronic databases will be searched: EMBASE (OvidSP), MEDLINE (OvidSP), Science Citation Index & Social Science Citation Index via Web of Science Core Collection, CINAHL (EBSCOHost), PsycINFO (OvidSP) and ASSIA (Proquest), and systematic review repositories: Cochrane Database of Systematic Reviews (Cochrane Library, Wiley) and Database of Abstracts of Reviews of Effects (https://www.crd.york.ac.uk/CRDWeb/). Grey literature sources will include backward and forward citation searches for all included articles and reference lists of related systematic reviews. Relevant third-sector organizations such as Birth Rights, Birth Companions, Maternity Action, and Sands will also be searched for evidence summaries, alongside the first 150 results of Google Scholar and clinicaltrials.gov.

The titles and abstracts of the studies retrieved during the searches will be independently assessed by two reviewers for relevance. The full texts of potentially eligible studies will then be retrieved and reviewed independently using a checklist of the inclusion and exclusion criteria by two reviewers. Any disagreements will be resolved by consensus and, if necessary, a third review researcher will be consulted, and will resolve issues. A table of excluded studies is found in Additional file 2.

A data extraction form will be designed in Excel and initially piloted on a small sample of papers by two review authors. Data extraction for the first 20% of the studies will be undertaken independently by two authors, if there is good agreement then a single author will complete data extraction for the remaining studies. This will include (1) author and publication year, (2) methodology (aim, type of review, location), (3) population characteristics (number of participants, demographics), (4) search methods (number of databases searched, date range of searching, publication date range of studies included in the review that inform each outcome of interest, (5) number of studies, type of studies, country of origin of studies included in each review, (6) instrument used to appraise the primary studies and their risk of bias, (7) included intervention details including timing, duration, setting (e.g., community or hospital) and mode of delivery, (8) outcomes related to this question, (9) method of synthesis and (10) quality of the included primary studies as assessed by review authors, (11) conflict of interest. In line with PRISMA guidelines, a flowchart will summarize the selection process. EndNote™ will be used to collect and manage the studies retrieved [30]. Covidence™ will be used for deduplication and study selection [31]. We will extract data from the systematic review (and any supplementary material), not from original primary studies. If there is data missing or inadequately described in reviews, we will note the gap in coverage but will not routinely refer back to the primary studies due to the additional burden on time and resources [29, 32].

The AMSTAR 2 appraisal tool will be used to assess the quality of included reviews [33]. This will enable an overall rating of confidence in the results of the review (high, moderate, low, or critically low). Quality appraisal results will be summarized in a table, including the rating for each question of the tool for each review, the rationale behind assessments, and the overall rating for each review. All reviews meeting the inclusion criteria will be presented, irrespective of AMSTAR findings [33]. Two authors will assess the quality of each text for 20% of studies with discrepancies resolved by consensus. If there is good agreement then a single author will assess the quality of the remaining texts.

### Data synthesis

This data synthesis plan is informed by Cochrane guidance for undertaking an overview of reviews [32]. Due to the broad topic being studied and the expected heterogeneity of the exposure group and interventions, we will present a narrative synthesis of the systematic reviews and not meta-analysis.

We will present an overlap of primary studies using a pairwise intersection heat map [34]. This has been chosen to visually and simply demonstrate patterns of high or low overlap given the anticipated large number of primary studies that will be included. Where meta-analysis from separate reviews contains overlapping primary studies, we will prioritize the summary result from the highest quality review according to the AMSTAR rating.

We will map the available evidence and explore the patterns in the data. For example, we will describe the findings according to the target population, for example, whether a specific social exposure (e.g., domestic abuse and intimate partner violence) was the focus or broader inclusion criteria were used and categorize interventions by the timing of delivery (pre-pregnancy, pregnancy or postnatal) and the level of change that was intended, for example individual, organizational or community interventions. Where there is sufficient homogeneity in the population, intervention type, and outcome, we will describe similarities and differences between the findings, taking into account the strength of evidence and risk of bias of the reviews and the included studies.

Since the purpose of the review is to identify the breadth and effectiveness of interventions, where relevant reviews entirely meet our inclusion criteria, we will summarize the narrative or meta-analysis results, reporting the summary result, 95% confidence intervals, and measures of heterogeneity [32]. In this case, we will extract and report GRADE assessments, where available, to describe the certainty of evidence. If relevant reviews include only some primary studies conducted in the target population/setting/study design, then the findings of these primary studies will be presented, where there is a specific subgroup reported or at least three primary studies meet our inclusion criteria. In this case, we will narratively re-synthesize the outcome data from primary studies that meet the inclusion criteria of this review (e.g., primary studies of pregnant women with social disadvantage in a review that included all pregnant women). Where GRADE assessments are available for these subgroups these will also be extracted and reported. Overall, we will then narratively describe interventions according to whether they are effective, promising, ineffective, or probably ineffective or unable to conclude effectiveness.

### Stakeholder engagement

To enhance the usefulness of the review we will actively involve stakeholders (families, midwives, doctors, health visitors, third sector, health decision-makers, and funders). We will create a lived experience team of women with social disadvantages around pregnancy and use a series of discussions to explore the acceptability and relevance of the identified interventions. We will carry out two stakeholder workshops to explore how the findings fit with current guidance, practice, and need, in the context of the UK health and social care system, in order to identify the implications for services, policy, and research.

## Discussion

The design of this umbrella review has been chosen to provide an overview of the range and efficacy of interventions aiming to mitigate social disadvantages around pregnancy. This design is beneficial in giving an overview of the types of interventions and quality of research in this field to date [35]. This is specifically useful given the aim of evaluating interventions that may work across health and social care systems before, during, and after pregnancy. However, there are limitations in this approach in that the heterogeneity in studies increases the burden for decision-makers [36] and promising interventions that have not been examined in systematic reviews will not be included.

This study is also limited to exploring the quantitative effect of interventions on health outcomes. This simple perspective was selected to provide a clear resource for clinicians and policymakers. However, it leaves many questions about the components of multifaceted interventions, and how they work and interact in different settings, unanswered [37, 38].

The findings of this umbrella review will enable recommendations for future research where there are gaps for women known to be at increased risk. We also hope to identify potential effective interventions that warrant further investigation, or that can inform policy and practice to reduce inequalities in maternal and child health.

### Supplementary Information

Additional file 1. MEDLINE search strategy.

Additional file 2. PRISMA-P 2015 Checklist.

## Data Availability

Data sharing is not applicable to this article as no datasets were generated or analyzed during the current study.

## References

[1] Knight M, Bunch K, Felker A, Patel R, Kotnis R, Kenyon S, Kurinczuk JJ, on behalf of MBRRACE-UK, editors. Saving lives, improving mothers’ care core report - lessons learned to inform maternity care from the UK and Ireland Confidential Enquiries into Maternal Deaths and Morbidity 2019–21. Oxford: National Perinatal Epidemiology Unit: University of Oxford; 2023. https://www.npeu.ox.ac.uk/assets/downloads/mbrrace-uk/reports/maternal-report-2023/MBRRACE-UK_Maternal_Compiled_Report_2023.pdf. Accessed 15 May 2024.

[2] Draper ES GI, Smith LK, Matthews RJ, Fenton AC, Kurinczuk JJ, Smith PW, Manktelow BN, on behalf of the MBRRACE-UK Collaboration. MBRRACE-UK perinatal mortality surveillance: UK perinatal deaths for births from January to December 2021: The State of the Nation Report Leicester: The Infant Mortality and Morbidity Studies: Department of Population Health Sciences, University of Leicester; 2023. https://timms.le.ac.uk/mbrrace-uk-perinatal-mortality/surveillance/. Accessed 15 May 2024.

[3] Jardine J, Walker K, Gurol-Urganci I, Webster K, Muller P, Hawdon J (2021). Adverse pregnancy outcomes attributable to socioeconomic and ethnic inequalities in England: a national cohort study. Lancet.

[4] Jutte DP, Brownell M, Roos NP, Schippers C, Boyce WT, Syme SL (2010). Rethinking what is important: biologic versus social predictors of childhood health and educational outcomes. Epidemiology.

[5] Houtepen LC, Heron J, Suderman MJ, Fraser A, Chittleborough CR, Howe LD (2020). Associations of adverse childhood experiences with educational attainment and adolescent health and the role of family and socioeconomic factors: a prospective cohort study in the UK. PLoS Med.

[6] Department of Health & Social Care. Maternity Disparities Taskforce: terms of reference. 2022. https://www.gov.uk/government/publications/maternity-disparities-taskforce-terms-of-reference/maternity-disparities-taskforce-terms-of-reference. Accessed 15 May 2024.

[7] Alderwick H, Dixon J (2019). The NHS long term plan. BMJ.

[8] NHS England. Equity and equality guidance for local maternity systems. NHS England; 2021. https://www.england.nhs.uk/wp-content/uploads/2021/09/C0734-equity-and-equality-guidance-for-local-maternity-systems.pdf. Accessed 15 May 2024.

[9] Ki-Moon B. Global Strategy for Women's and Children's Health. New York: World Health Organisation: United Nations; 2010. https://www.ohchr.org/sites/default/files/Documents/Issues/Women/WRGS/Health/GlobalStrategy.pdf. Accessed 15 May 2024.

[10] National Institute for Health and Care Excellence. Pregnancy and complex social factors: a model for service provision for pregnant women with complex social factors. NICE guidelines CG110: National Institute for Health and Care Excellence; 2010. Available from: https://www.nice.org.uk/guidance/cg110. Accessed 15 May 2024. 31855334

[11] National Institute for Health and Care Excellence. Surveillance proposal consultation document: 2018 surveillance of pregnancy and complex social factors NICE: National Institute for Health and Care Excellence; 2018. https://www.nice.org.uk/guidance/cg110/resources/2018-surveillance-of-pregnancy-and-complex-social-factors-a-model-for-service-provision-for-pregnant-women-with-complex-social-factors-nice-guideline-cg110-6532362253/chapter/Overview-of-2018-surveillance-methods?tab=evidence. Accessed 15 May 2024.

[12] Stewart E, Pearce A, Given J, Gilbert R, Brophy S, Cookson R (2023). Identifying opportunities for upstream evaluations relevant to child and maternal health: a UK policy-mapping review. Arch Dis Child.

[13] Conry JA (2023). Women's health across the life course and opportunities for improvement: Every woman, every time, everywhere. Int J Gynecol Obstet..

[14] Khan Z, Vowles Z, Fernandez Turienzo C, Barry Z, Brigante L, Downe S (2023). Targeted health and social care interventions for women and infants who are disproportionately impacted by health inequalities in high-income countries: a systematic review. Int J Equity Health.

[15] Hollowell J, Oakley L, Kurinczuk JJ, Brocklehurst P, Gray R (2011). The effectiveness of antenatal care programmes to reduce infant mortality and preterm birth in socially disadvantaged and vulnerable women in high-income countries: a systematic review. BMC Pregnancy Childbirth.

[16] Pedersen JF, Kallesoe SB, Langergaard S, Overgaard C (2021). Interventions to reduce preterm birth in pregnant women with psychosocial vulnerability factors-A systematic review. Midwifery.

[17] Knight MBK, Patel R, Shakespeare J, Kotnis R, Kenyon S, Kurinczuk JJ, on behalf of MBRRACE-UK, editors. Lessons leaned to inform maternity care from the UK and Ireland Confidential Enquiries into Maternal Deaths and Morbidity 2018–20. Oxford: National Perinatal Epidemiology Unit: University of Oxford; 2022. https://www.npeu.ox.ac.uk/assets/downloads/mbrrace-uk/reports/maternal-report-2022/MBRRACE-UK_Maternal_MAIN_Report_2022_UPDATE.pdf. Accessed 15 May 2024.

[18] Peace R. Social exclusion: a concept in need of definition. Soc Policy J N Z. 2001;16:17–36. https://www.msd.govt.nz/documents/about-msd-and-our-work/publications-resources/journals-and-magazines/social-policy-journal/spj16/16-pages17-36.pdf. Accessed 17 May 2024.

[19] Fitzpatrick S, Bramley G, Johnsen S (2013). Pathways into multiple exclusion homelessness in seven UK cities. Urban Studies.

[20] Kuh D, Ben-Shlomo Y, Lynch J, Hallqvist J, Power C (2003). Life course epidemiology. J Epidemiol Community Health.

[21] NHS England. Core20PLUS5 (adults) - an approach to reducing healthcare inequalities. 2023 [cited 2023 August ]. Available from: https://www.england.nhs.uk/about/equality/equality-hub/national-healthcare-inequalities-improvement-programme/core20plus5/.

[22] Shamseer L, Moher D, Clarke M, Ghersi D, Liberati A, Petticrew M (2015). Preferred reporting items for systematic review and meta-analysis protocols (PRISMA-P) 2015: elaboration and explanation. BMJ.

[23] Gribble KD, Bewley S, Bartick MC, Mathisen R, Walker S, Gamble J (2022). Effective communication about pregnancy, birth, lactation, breastfeeding and newborn care: the importance of sexed language. Front Glob Women's Health..

[24] Sangsawang B, Wacharasin C, Sangsawang N (2019). Interventions for the prevention of postpartum depression in adolescent mothers: a systematic review. Arch Womens Ment Health.

[25] Harden A, Brunton G, Fletcher A, Oakley A (2009). Teenage pregnancy and social disadvantage: systematic review integrating controlled trials and qualitative studies. BMJ.

[26] Bottorff JL, Poole N, Kelly MT, Greaves L, Marcellus L, Jung M (2014). Tobacco and alcohol use in the context of adolescent pregnancy and postpartum: a scoping review of the literature. Health Soc Care Community.

[27] Chow R, Huang E, Li A, Li S, Fu SY, Son JS (2021). Appraisal of systematic reviews on interventions for postpartum depression: systematic review. BMC Pregnancy Childbirth.

[28] Esan OBAN, Saberian S, Christianson L, McHale P, Pennington A, Geary R, Ayorinde A. Mapping existing policy interventions to tackle ethnic health inequalities in maternal and neonatal health in England: a systematic scoping review with stakeholder engagement. NHS Race & Health Observatory; 2022. https://www.nhsrho.org/wp-content/uploads/2022/12/RHO-Mapping-existing-policy-interventions_December-2022.pdf. Accessed 15 May 2024.

[29] Aromataris E, Fernandez R, Godfrey CM, Holly C, Khalil H, Tungpunkom P (2015). Summarizing systematic reviews: methodological development, conduct and reporting of an umbrella review approach. Int J Evid Based Healthc.

[30] Endnote (2013). EndNote.

[31] Veritas (2021). Covidence Systematic Review Software.

[32] Pollock M, Fernandes RM, Becker LA, Pieper D, Hartling L. Chapter V: Overviews of Reviews. In Higgins JPT, Thomas J, Chandler J, Cumpston M, Li T, Page MJ, Welch VA, editors. Cochrane; 2023. Accessed https://training.cochrane.org/handbook/current/chapter-v. Accessed 15 May 2024.

[33] Shea BJ, Reeves BC, Wells G, Thuku M, Hamel C, Moran J (2017). AMSTAR 2: a critical appraisal tool for systematic reviews that include randomised or non-randomised studies of healthcare interventions, or both. BMJ.

[34] Bougioukas KI, Vounzoulaki E, Mantsiou CD, Savvides ED, Karakosta C, Diakonidis T (2021). Methods for depicting overlap in overviews of systematic reviews: An introduction to static tabular and graphical displays. J Clin Epidemiol.

[35] Lazaros B, Vanesa B, John PAI (2022). Conducting umbrella reviews BMJ Medicine.

[36] Higgins JPT, Thomas J, Chandler J, Cumpston M, Li T, Page MJ, Welch VA, editors. Cochrane Handbook for Systematic Reviews of Interventions. The Cochrane Collaboration, editor. John Wiley & Sons; 2011. https://training.cochrane.org/handbook/current. Accessed 15 May 2024.

[37] Petticrew M, Anderson L, Elder R, Grimshaw J, Hopkins D, Hahn R (2013). Complex interventions and their implications for systematic reviews: a pragmatic approach. J Clin Epidemiol.

[38] Julian PTH, José AL-L, Betsy JB, Sarah RD, Sarah D, Jeremy MG (2019). Synthesising quantitative evidence in systematic reviews of complex health interventions. BMJ Global Health..

