# Supplementary material for: Interventions to reduce inequalities for pregnant women living with disadvantage in high-income countries: an umbrella review protocol
Source: Syst Rev. 2024 May 23;13:139. doi: 10.1186/s13643-024-02556-7 (PMC11112858; doi:10.1186/s13643-024-02556-7)
Supplement: Supplementary file 1 — Additional file 1. MEDLINE search strategy. [file 13643_2024_2556_MOESM1_ESM.docx]

**Medline search strategy**

|  | [Medline (Ovid MEDLINE® Epub Ahead of Print, In-Process & Other Non-Indexed Citations, Ovid MEDLINE® Daily and Ovid MEDLINE®) 1946 to present](https://ovidsp.ovid.com/ovidweb.cgi?T=JS&NEWS=N&PAGE=main&SHAREDSEARCHID=42KZG6wV5VYVXpeZUgOn1iMiAJ39KJD4jN1dEvu9sB3mj26Z5mRNxMbpWRpRwrZRm) |
| --- | --- |
| 1 | intersectional framework/ |
| 2 | Socioeconomic Factors/ |
| 3 | Vulnerable Populations/ or Minority Groups/ |
| 4 | social vulnerability/ |
| 5 | sociodemographic factors/ |
| 6 | Healthcare Disparities/ |
| 7 | economic factors/ or economic stability/ or housing instability/ or exp ill-housed persons/ or economic status/ or poverty/ or poverty areas/ or social class/ or low socioeconomic status/ or social mobility/ or social factors/ or working poor/ |
| 8 | social isolation/ |
| 9 | domestic violence/ or intimate partner violence/ or spouse abuse/ or battered women/ |
| 10 | exp educational status/ or employment/ or unemployment/ |
| 11 | sex workers/ or sex work/ |
| 12 | human traffiking/ or enslaved persons/ |
| 13 | crime/ or criminals/ or prisoners/ or criminal law/ or prisons/ |
| 14 | deportation/ or refugees/ or exp "emigrants and immigrants"/ |
| 15 | drug users/ |
| 16 | exp indigenous peoples/ or roma/ or medically uninsured/ or exp asian people/ or exp black people/ or caribbean people/ |
| 17 | (socioeconomic? or socio-economic? or sociodemographic? or socio-demographic? or psychosocial or psycho-social).ti,kf. |
| 18 | ((socioeconomic? or socio-economic? or sociodemographic? or socio-demographic? or psychosocial or psycho-social or social or economic) adj3 (factor? or risk? or status or level?)).ab. |
| 19 | ((social or economic) adj3 (disparit* or disadvantag* or vulnerab* or depriv* or hardship)).ti,ab,kf. |
| 20 | (social* adj2 isolat*).ti,ab,kf. |
| 21 | social determinants of health.ti,ab,kf. |
| 22 | ((disadvantag* or vulnerab* or depriv* or hardship) adj3 (wom?n or population? or people or communit* or neighbo?rhood? or area? or family or families)).ti,ab,kf. |
| 23 | ((poor or poverty) adj3 (wom?n or population? or people or communit* or neighbo?rhood? or area? or family or families)).ti,ab,kf. |
| 24 | (homeless* or couchsurf* or couch surf* or (hous* adj3 (instability or unstab* or uncertain*))).ti,ab,kf. |
| 25 | (income adj3 (low* or level? or status)).ti,ab,kf. |
| 26 | (((education* or academic) adj3 (level? or status or achievement or attainment)) or (numeracy or literacy)).ti,ab,kf. |
| 27 | ((spous* or domestic or intimate partner) adj2 (abuse* or violence)).ti,ab,kf. |
| 28 | (sex work* or prostitut*).ti,ab,kf. |
| 29 | (modern slave* or (enslav* adj2 (worker? or people or person? or population? or wom?n))).ti,ab,kf. |
| 30 | (criminal* or prison* or incarcerat* or imprison* or probation* or detention centre? or detention center? or jail? or gaol?).ti,ab,kf. |
| 31 | **(refugee? or asylum seeker? or migrant? or immigrant? or deportation).ti,ab,kf.** |
| 32 | ((black? or afrocarib* or afro-carib* or african american or latino? or latina? or latinix or hispanic or asian or indigenous or aborigin* or roma or gypsy or traveller) adj2 (people or person or population or wom?n or family or families or communit*)).ti,ab,kf. |
| 33 | (ethnic* or minorit* or race or racial).ti,kf. |
| 34 | ((ethnic* or minorit* or racial) adj2 (group? or people or person or population or wom?n)).ab. |
| 35 | (complex* adj3 (need? or risk? or factor?)).ti,ab,kf. |
| 36 | or/2-35 |
| 37 | Pregnancy/ |
| 38 | Pregnant Women/ |
| 39 | (pregnan* or antenatal or ante-natal or prenatal or pre-natal or perinatal or peri-natal or peripart* or peri-part* or intrapart* or intra-part* or postnatal or post-natal or postpart* or post-part* or maternal).ti,kf. |
| 40 | (expectant wom?n or expectant mother? or pregnant wom?n).ti,ab,kf. |
| 41 | or/37-40 |
| 42 | maternal health services/ or maternal-child health services/ or perinatal care/ or postnatal care/ or prenatal care/ |
| 43 | maternal-child nursing/ or midwifery/ |
| 44 | health education/ or health promotion/ or patient education as topic/ or prenatal education/ or smoking prevention/ |
| 45 | Counseling/ |
| 46 | House Calls/ |
| 47 | Early Medical Intervention/ |
| 48 | Social Support/ |
| 49 | (program* or service? or clinic? or intervention?).ti. |
| 50 | ((pregnan* or antenatal or ante-natal or prenatal or pre-natal or perinatal or peri-natal or peripart* or peri-part* or intrapart* or intra-part* or postnatal or post-natal or postpart* or post-part* or maternal or midwi*) adj3 (program* or service? or clinic? or intervention?)).ti,ab,kf. |
| 51 | ((pregnan* or antenatal or ante-natal or prenatal or pre-natal or perinatal or peri-natal or peripart* or peri-part* or intrapart* or intra-part* or postnatal or post-natal or postpart* or post-part* or maternal) adj3 education*).ti,ab,kf. |
| 52 | ((pregnan* or antenatal or ante-natal or prenatal or pre-natal or perinatal or peri-natal or peripart* or peri-part* or intrapart* or intra-part* or postnatal or post-natal or postpart* or post-part* or maternal or midwi*) adj3 (care or healthcare)).ti,kf. |
| 53 | ((pregnan* or antenatal or ante-natal or prenatal or pre-natal or perinatal or peri-natal or peripart* or peri-part* or intrapart* or intra-part* or postnatal or post-natal or postpart* or post-part* or maternal or midwi*) adj3 (model? or system?)).ti,ab,kf. |
| 54 | ((communit* or neighbo?rhood?) adj3 (program* or service? or intervention?)).ti,ab,kf. |
| 55 | ((behav* or risk reduction) adj3 (program* or service? or intervention?)).ti,ab,kf. |
| 56 | ((diet* or nutrition* or obes* or weight*) adj3 (program* or service? or intervention?)).ti,ab,kf. |
| 57 | ((smok* or tobacco or alcohol or drink* or substance or drug?) adj3 (program* or service? or intervention?)).ti,ab,kf. |
| 58 | ((health adj3 (education* or promotion)) or patient education).ti,ab,kf. |
| 59 | ((pregnan* or antenatal or ante-natal or prenatal or pre-natal or perinatal or peri-natal or peripart* or peri-part* or intrapart* or intra-part* or postnatal or post-natal or postpart* or post-part* or maternal) adj3 (counsel* or behavi* therap* or cognitive therap*)).ti,ab,kf. |
| 60 | or/42-59 |
| 61 | fetal mortality/ or infant mortality/ or perinatal mortality/ or maternal mortality/ |
| 62 | Pregnancy Outcome/ or Outcome Assessment, Health Care/ or "Outcome and Process Assessment, Health Care"/ |
| 63 | exp Pregnancy Complications/ |
| 64 | exp infant, low birth weight/ or exp infant, premature/ or Birth Weight/ or Gestational Age/ |
| 65 | Breast Feeding/ |
| 66 | Depression/pc or Anxiety/pc or Depressive Disorders/pc or Anxiety Disorders/pc |
| 67 | health services accessibility/ or access to primary care/ or health equity/ |
| 68 | help-seeking behavior/ |
| 69 | social inclusion/ |
| 70 | exp "Patient Acceptance of Health Care"/ |
| 71 | ((preterm* or prematur*) adj3 (birth? or chilbirth? or labo?r or infant?)).ti,ab,kf. |
| 72 | (onset adj3 (birth? or chilbirth? or labo?r or infant?)).ti,ab,kf. |
| 73 | (miscarriage? or spontanous abortion? or stillbirth? or still birth? or ((fetal or foetal or fetus or foetus) adj (death? or mortality))).ti,ab,kf. |
| 74 | (birthweight or birth weight).ti,kf. or (low adj (birthweight or birth weight)).ab. or gestational age.ti,kf. or "small for gestational age".ab. |
| 75 | ((pregnancy or maternal or neonat* or newborn? or infant?) adj3 (outcome? or death? or mortality or complication?)).ti,ab,kf. |
| 76 | (caesarean? or cesarean? or ((assisted or surg*) adj2 deliver*)).ti,ab,kf. |
| 77 | ((intensive care or nicu or icu or itu) adj3 (admission? or admit* or transfer*)).ti,ab,kf. |
| 78 | (breastfeeding or breast feeding).ti,ab,kf. |
| 79 | (depress* or mood disorder?).ti,ab,kf. |
| 80 | (access* adj5 (service? or care or healthcare)).ti,ab,kf. |
| 81 | (continuity* adj3 (service? or care or healthcare)).ti,ab,kf. |
| 82 | ((engag* or attend*) adj3 (clinic? or service? or program*)).ti,ab,kf. |
| 83 | (((health* or care) adj (equit* or inequit* or equalit* or inequalit* or disparit*)) or social inclusion).ti,ab,kf. |
| 84 | ((health* or care or help) adj2 seek*).ti,ab,kf. |
| 85 | or/61-84 |
| 86 | 1 and 41 |
| 87 | 36 and 41 and 60 and 85 |
| 88 | 86 or 87 |
| 89 | afghanistan/ or exp africa/ or albania/ or andorra/ or antarctic regions/ or argentina/ or exp asia, central/ or exp asia, northern/ or exp asia, southeastern/ or exp atlantic islands/ or bahrain/ or bangladesh/ or bhutan/ or bolivia/ or borneo/ or "bosnia and herzegovina"/ or brazil/ or bulgaria/ or exp central america/ or exp china/ or colombia/ or "commonwealth of independent states"/ or croatia/ or "democratic people's republic of korea"/ or ecuador/ or gibraltar/ or guyana/ or exp india/ or indonesia/ or iran/ or iraq/ or jordan/ or kosovo/ or kuwait/ or lebanon/ or liechtenstein/ or macau/ or "macedonia (republic)"/ or exp melanesia/ or moldova/ or monaco/ or mongolia/ or montenegro/ or nepal/ or netherlands antilles/ or new guinea/ or oman/ or pakistan/ or paraguay/ or peru/ or philippines/ or qatar/ or "republic of belarus"/ or romania/ or exp russia/ or saudi arabia/ or serbia/ or sri lanka/ or suriname/ or syria/ or taiwan/ or exp transcaucasia/ or ukraine/ or uruguay/ or united arab emirates/ or exp ussr/ or venezuela/ or yemen/ |
| 90 | organisation for economic co-operation and development/ |
| 91 | australasia/ or exp australia/ or austria/ or exp baltic states/ or belgium/ or exp canada/ or chile/ or czech republic/ or europe/ or exp france/ or exp germany/ or greece/ or hungary/ or ireland/ or israel/ or exp italy/ or exp japan/ or korea/ or luxembourg/ or mexico/ or netherlands/ or new zealand/ or north america/ or poland/ or portugal/ or exp "republic of korea"/ or exp "scandinavian and nordic countries"/ or slovakia/ or slovenia/ or spain/ or switzerland/ or turkey/ or exp united kingdom/ or exp united states/ |
| 92 | european union/ |
| 93 | developed countries/ |
| 94 | 90 or 91 or 92 or 93 |
| 95 | 89 not 94 |
| 96 | 88 not 95 |
| 97 | limit 96 to yr="2013 -Current" |
| 98 | limit 97 to (meta analysis or systematic review or "reviews (maximizes specificity)") |
| 99 | (comment or editorial or letter or news).pt. |
| 100 | 98 not 99 |
